# Effects of Dietary *Callicarpa nudiflora* Aqueous Extract Supplementation on Growth Performance, Growth Hormone, Antioxidant and Immune Function, and Intestinal Health of Broilers

**DOI:** 10.3390/antiox13050572

**Published:** 2024-05-06

**Authors:** Mengjie Liu, Gengxiong Huang, Yulin Lin, Yiwen Huang, Zhaoying Xuan, Jianchi Lun, Shiqi He, Jing Zhou, Xiaoli Chen, Qian Qu, Weijie Lv, Shining Guo

**Affiliations:** 1 College of Veterinary Medicine, South China Agricultural University, Guangzhou 510642, China; 2 Guangdong Technology Research Center for Traditional Chinese Veterinary Medicine and Nature Medicine, Guangzhou 510642, China; 3 International Institute of Traditional Chinese Veterinary Medicine, Guangzhou 510642, China

**Keywords:** *Callicarpa nudiflora* aqueous extract, growth performance, antioxidant function, immunity, intestinal health

## Abstract

*C. nudiflora* is notably rich in flavonoids and phenylethanoid glycosides, making it a significant natural source of antioxidants. We examined the effects of *C. nudiflora* aqueous extract (CNE) on growth performance, antioxidant function, immunity, intestinal barrier function, nutrient transporters, and microbiota of broilers. A total of 360 one-day-old broilers were randomly assigned to four treatment groups: a basal diet with 0 (control, CON), 300 mg/kg (CNEL), 500 mg/kg (CNEM), and 700 mg/kg (CNEH) CNE for 42 days. CNEL and CNEM groups quadratically increased body weight and average daily gain but decreased feed-to-gain ratios during the starter and whole phases. Regarding the immune response of broilers, CNE treatment linearly down-regulated jejunal myeloid differentiation factor 88 (MyD88) expression and interleukin-1β (IL-1β) and interferon-γ expression in the liver (d 21), while decreasing jejunal IL-1β expression and the concentration of serum tumor necrosis factor-α and interleukin-6 (d 42). The CNEM and CNEH groups had lower MyD88 and nuclear factor kappa B expression in the liver (d 21) compared to the CON group. Broilers in the CNEL and CNEM groups had higher spleen index and thymus index (d 21) and interleukin-10 expression from the liver and jejunal mucosa (d 42) than that in the CON group. For the antioxidant capacity of broilers, CNE treatment linearly decreased the content of malonaldehyde and increased the activity of total antioxidant capacity in serum (d 42). CNEM and CNEH groups linearly increased the activity of superoxide dismutase in serum and heme oxygenase-1 expression in the liver, while increasing the activity of glutathione peroxidase in serum, jejunal nuclear factor E2-related factor 2 expression, and NAD(P)H quinone oxidoreductase 1 expression in the liver (d 42). As for the growth hormone of broilers, CNEM group increased the level of serum insulin-like growth factor 1 and up-regulated jejunal glucagon-like peptide 2 (GLP-2) expression (d 21). Broilers in the CNEM and CNEH groups had higher jejunal GLP-2 expression and growth hormone (GH) expression in the liver and the level of serum GH (d 42) than that in the CON group. Additionally, the villus height and jejunal Occludin and Claudin-1 expression in the CNEM group increased. CNE-containing diets resulted in a linear increase in the expression of jejunal zonula occluden-1 (d 21), villus height to crypt depth ratio, jejunal Occludin, excitatory amino acid transporters-3, and peptide-transporter 1 (d 42). The regulation of *Oscillospira*, *Ruminococcaceae_Ruminococcus*, and *Butyricicoccus* genera indicated that CNEH altered the composition of the cecal microbiota. In general, supplementing broilers with *C. nudiflora* aqueous extract could boost hormones, immune and antioxidant function, and gut health, improving their growth performance. Hence, CNE was a promising poultry feed additive, with 500 mg/kg appearing to be the optimal dose.

## 1. Introduction

The growth and development of poultry are hindered by increasing challenges associated with intensive production, such as pathogenic germs, environmental factors, and feed hygiene [1,2]. However, the widespread use of antibiotics for disease prevention and growth promotion poses several issues, including environmental contamination and concerns about food safety [3]. Numerous countries, including the USA and the European Union, have enacted laws restricting or completely prohibiting the use of antibiotics in the feed of farm animals [4]. China stopped producing commercial feed with feed additives containing growth-promoting drugs on 1 July 2020, with the exception of traditional Chinese medicine [1]. Therefore, it was crucial to develop in-feed antibiotic substitutes that effectively enhance animal productivity without compromising human health. Recent studies have demonstrated the benefits of natural plant extracts, including traditional herbal remedies, which are biologically active and free from residues. In the production of chickens, these substances are frequently added to the feed [5]. Because they are natural plant products with antibacterial, antioxidant, and immunomodulatory qualities, herbal extracts and TCM (Traditional Chinese Medicine) have become intriguing substitutes [6].

*Callicarpa nudiflora* Hook, a member of the family Verbenaceae and phylum *Callicarpa*, is found in large numbers in the Guangdong, Guangxi, and Hainan regions of China [7]. Modern pharmacological studies have shown that the isolated components or extracts of *Callicarpa* demonstrate anti-inflammatory, hemostatic, antioxidant, antibacterial, and analgesic properties [8]. Based on available information, *C. nudiflora Hook* primarily consists of phenylethanol glycosides, flavonoids, iridoid glycosides, volatile oils, triterpenes, and diterpenes [9]. Verbascoside, also called kusaginin and acteoside, is a naturally occurring, water-soluble phenylpropanoid glycoside [10]. Verbascoside exhibits several properties, including antioxidant, anti-inflammatory, chemopreventive, and neuroprotective properties, which improves livestock health, antioxidant status, and product quality [11]. According to Xiao et al. [12], rats receiving 100 mg/kg VB orally had a maximum serum concentration of 0.13 μg/mL, a half-life of 92.1 min, and a bioavailability of 0.12%. Additionally, the capacity of VB to chelate metal ions was a determining factor in the antioxidant activity of natural extracts containing VB (485 mg/100 g dry weight) [13]. In vitro studies show that verbascoside (22 μg/mL) extracted from olive oil has antioxidant effects in HT-29 cells, preventing lipid peroxidation and directly scavenging physiologically active free radicals [14]. In addition, *C. nudiflora* extract (100 mg/kg) lowers inflammatory cytokine levels in H1N1-infected mouse serum and reduces lung tissue damage, demonstrating anti-inflammatory properties. Zhang et al. [15] showed that the anti-inflammatory effects of *C. nudiflora* alcohol extract (100 mg/kg) are demonstrated by a decrease in the amount of inflammatory cytokines (IL-6, TNF-α, IL-1β) in the serum of H1N1-infected mice and a reduction in lung tissue damage. Ma et al. [16] reported that oral administration of *Callicarpa nudiflora* extract (0.15 and 0.3 g/kg) in streptozotocin-induced diabetic rats improved insulin resistance and reversed diabetes damage to the liver and pancreas. Moreover, luteolin is a common flavonoid extracted from *Callicarpa nudiflora* [17]. Lutein possesses a variety of pharmacological properties due to its ability to regulate the redox state, and its properties also include anti-inflammatory, antibacterial, and neuroprotective activities [18]. Several studies on anti-inflammatory effects have reported that luteolin (20 mg/kg) can prevent diabetic cardiomyopathy by inhibiting NF-κB-mediated inflammation and inducing Nrf2-mediated antioxidant responses [19]. Furthermore, when fed mice were exposed to lipopolysaccharides (LPSs), luteolin at 100 mg/kg significantly protected them against oxidative stress and liver damage [20]. The results of these studies might further support the potential application of *C. nudiflora* with antioxidant effects in the production and diets of broilers.

A healthy and well-developed gut in chickens is necessary for proper absorption and digestion as well as defense against infections [21]. In particular, the microbiota play a critical role in intestinal formation, as well as in immunological and physiological processes [22]. Prior studies have demonstrated the numerous beneficial effects of flavonoids, including enhanced immunity and improved gut architecture and function [23]. Flavonoids possess the capacity to modify the makeup of the gut microbiome, impede the proliferation of specific pathogens, and stimulate the growth of advantageous bacterial genera like *Lactobacillus* [24]. Thus, the potential interactions of *Callicarpa nudiflora* extract on the microbiota and function of the intestinal barrier of broilers need to be further studied.

In many nations (including China), *Callicarpa nucifora* Hook is also permitted to be used as an additive in feed for breeding livestock and poultry. Nevertheless, a thorough evaluation of the impact and strategies for attaining the growth-promoting effect has not been conducted. Therefore, the aim of this study was to examine the effects of supplementing *C. nudiflora* aqueous extract on growth performance, antioxidant capacity, immune response, growth hormone level, mucosal barrier function, nutrient transporters, and cecal microbiota of broilers, as well as to provide a reference for the use of *C. nudiflora* aqueous extract in the poultry industry.

## 2. Materials and Methods

### 2.1. Callicarpa nudiflora Water Extraction and Chemical Components Determination

The powder of *Callicarpa nudiflora* leaves was obtained from Guangzhou Geleite Biotechnology Co., Ltd. (Guangdong, China). With some modifications, the materials were produced and examined following previously reported methodologies [25]. In brief, a total of 200 g of *Callicarpa nudiflora* powder was weighed and steeped in water at a 10-fold dilution for 30 min and, then, boiled for 2 h (100 °C). After filtering, the mixture was heated for an additional hour (90 °C) before being filtered again, and water was then included to produce an eight-fold dilution. Following filtration, the mixture was concentrated. Subsequently, the extract was freeze-dried, and the yield rate was 15.49%.

**Figure 1:**
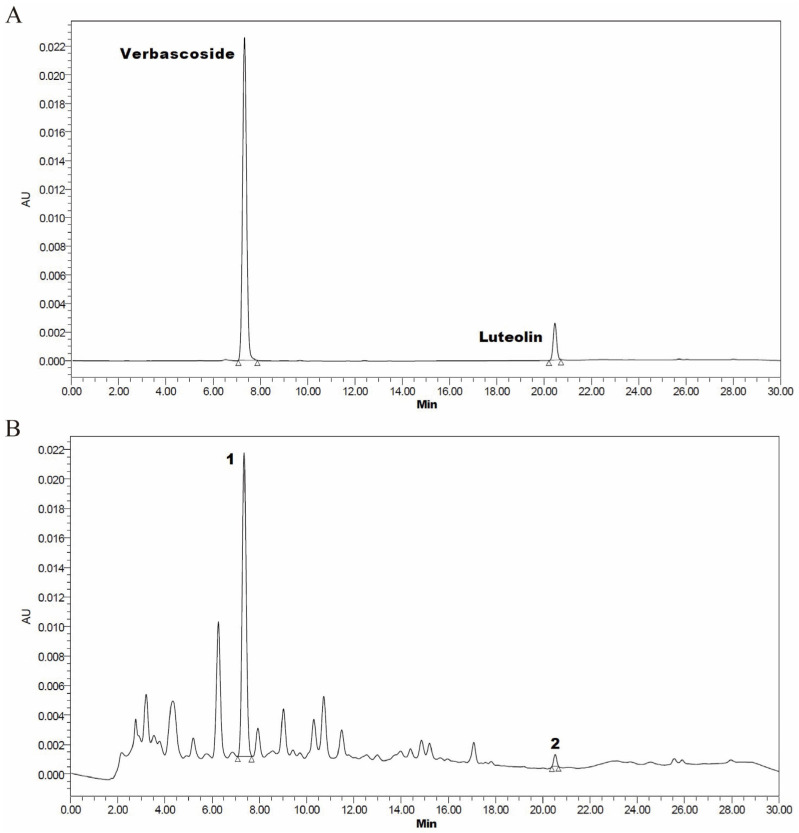
The chromatogram of standard product and test product of verbascoside and luteolin. (**A**) Verbascoside and luteolin standard product. (**B**) *Callicarpa nudiflora* aqueous extract (CNE), (1) verbascoside, (2) luteolin.

Verbascoside (CAS: 61276-17-3) and luteolin (CAS: 491-70-3) (purity ≥ 98%) were purchased from Chengdu Herbpurify Co., Ltd. (Chengdu, China). Samples were examined using high performance liquid chromatography systems (Waters, Milford, MA, USA) in accordance with the published protocol with minor adjustments [26,27]. The condition of HPLC was as follows: LC: waters 2489 HPLC; Column: Agilent C18 (4.6 mm × 250 mm, 5 μm); Column Temperature: 30 °C; Injection Volume: 10 µL; mobile phase: A: 0.3% phosphoric acid aqueous solution; B: acetonitrile. The gradient elution procedure is shown in Supplemental Table S1. UV spectra were recorded at 350 nm. The contents of verbascoside and luteolin in CNE were 23.38 mg/g and 0.33 mg/g by high performance liquid chromatography (HPLC) (Figure 1).

The total phenol content and flavonoid content in the powder of *C. nudiflora* aqueous extract were tested using the commercial kits from Nanjing Jiancheng Bioengineering Institute (Nanjing, China) with a Microplate reader (InterMed, South Portland, ME, USA). The kit information is listed in Supplemental Table S2. The total phenol content was measured at 760 nm based on the phenolic substance that reduced the tungsten molybdic acid to produce a somewhat blue compound in an alkaline nitrite solution, in accordance with the published protocol by Wang et al. [28]. The flavonoid content was determined at 502 nm using a method where flavonoids and aluminum ions form a red complex under alkaline conditions [28]. The contents of total phenols and flavonoids in CNE were 54.89 mg/g and 6.27 mg/g, respectively.

### 2.2. Birds, Experimental Design, and Diets

A total of 360 one-day-old male white-feathered broilers (34.6 ± 2.3 g) were purchased from Foshan City Gaoming District Xinguang Agriculture and Animal Husbandry Co., Ltd. (Guangdong, China). The experiment birds were split into four groups starting from day 1 of age (15 chicks/replicate × 6 replicates/group): the control group without any addition (CON); and three experimental groups fed a basal diet supplemented with 300 mg/kg (CNEL), 500 mg/kg (CNEM), and 700 mg/kg (CNEH) of *Callicarpa nudiflora* aqueous extract (CNE), respectively, for 42 days. The basal diet (Table S3) was formulated with reference to the nutritional requirements of broilers in the Chicken Feeding Standard (NY/T33-2004, Beijing, China) and NRC (1994) [29]. The broilers were housed in 3-layer cages with a 23-light:1-dark lighting routine and free access to feed and clean water throughout the experimental feeding period. The room temperature was maintained at 35 °C for 1 week of age and, then, gradually reduced to 20 °C until the end of the 42-day feeding experiment.

### 2.3. Growth Performance

The broiler body weight (BW) and feed intake were recorded on days 1, 21, and 42 of the experiment in order to calculate the average daily gain (ADG) and average daily feed intake (ADFI) values. The ADFI and ADG were utilized to calculate the feed-to-gain ratio (F/G) for every trial phase.

### 2.4. Sample Collection

After fasting for 12 h, one randomly selected broiler out of a replicate was sacrificed in the morning at 21 and 42 days of age. Blood samples were collected from a vein in the left wing of the broiler, centrifuged at 3000× *g* for 10 min at 4 °C, and stored at −20 °C for biochemical analysis. The birds were euthanized and slaughtered by cervical dislocation in the dissection room of the Laboratory Animal Centre of South China Agricultural University (Guangzhou, China). Following the opening of the birds, the lymphoid organs, including the thymus, bursa of Fabricius, and spleen, were isolated and weighed. The visceral weight index was calculated using the formula: visceral weight index (%) = (viscera weight/final body weight) × 100. After the small intestine was separated from the mesentery, the middle segments of the jejunum were collected (about 8 cm), gently washed with 5 mL of sterile saline, and fixed in 4% paraformaldehyde (about 2 cm) for paraffin embedding. The mid-jejunum mucosa was carefully scraped with a scalpel, and the liver was quickly excised, immediately placed in liquid nitrogen and stored at −80 °C for further analysis. The cecal contents were quickly collected in 2 mL cryogenic vials, rapidly preserved at −80 °C for additional microbiological examination after being frozen in liquid nitrogen.

### 2.5. Determination of Serum Growth Hormone and Immune and Antioxidant Parameters

The levels of growth hormone (GH), insulin-like growth factor-1 (IGF-1), immunoglobulin A (IgA), immunoglobulin M (IgM), interleukin-6 (IL-6), interleukin-10 (IL-10), and tumor necrosis factor-α (TNF-α) in the serum were determined using assay kits from Shanghai Enzyme-linked Biotechnology Co., Ltd. (Shanghai, China). The concentration of malondialdehyde (MDA) and the activity of glutathione peroxidase (GSH-Px), superoxide dismutase (SOD), and total antioxidant capacity (T-AOC) in the serum were measured using commercial assay kits from Nanjing Jiancheng Bioengineering Institute (Nanjing, China), following the manufacturer’s instructions. For the analysis, a V1600 Split Beam Visible Spectrophotometer (Meipuda Co., Shanghai, China) was utilized. The kit information is listed in Supplemental Table S2.

### 2.6. Measurements of Jejunal Morphology

The jejunum segment, after removal from the 4% paraformaldehyde fixing solution, was embedded in paraffin and sliced using a microtome. These paraffin slices were subsequently stained with hematoxylin and eosin (H&E staining). Refer to the prior study for details on the H&E staining procedures [30]. The microscope image processing application Image-Pro Plus 6.0 (Media Cybernetics, Rockville, MD, USA) was used to analyze the morphological features of the jejunum. The morphological data, including villus height (VH), crypt depth (CD), and the ratio of villus height to crypt depth (VH/CD), were computed using the acquired images.

### 2.7. Tissue RNA Extraction and qRT-PCR Analysis

The trial followed the established procedures of Liu et al. [31].Total RNA was extracted from the jejunal mucosa and liver samples using TRIzol reagent (Vazyme Biotech Co., Ltd., Nanjing, China). HiScript III RT SuperMix for qPCR (+gDNA wiper) (Vazyme) was used to synthesize total cDNA with total RNA (1 μg). Then, using the ChamQ universal SYBR qPCR Master Mix (Vazyme) in a QuantStudio^®^5 (Thermo Fisher Scientific, Inc., Waltham, MA, USA), real-time quantitative PCR (RT-qPCR) amplification was performed. The primers for immune-related (IL-1β, IL-6, IL-10, IFN-γ, TLR4, Myd88, and NF-κB), antioxidant-related (CAT, GSH-Px, SOD1, Nrf2, HO-1, and NQO1), growth-hormone-related (IGF-2, GH, and GLP2), tight junction protein (Claudin-1, Occludin, and ZO-1), and nutrient transporter (EAAT3, GLUT2, and PepT1) genes were designed with Primer Premier 6.0 software (Premier Biosoft International, Palo Alto, CA, USA) and synthesized by Tsingke Biotechnology Co., Ltd. (Beijing, China). The PCR primer sequences utilized in this investigation are indicated in Supplemental Table S4. Following normalization against the geometric mean of β-actin expression, the relative expression of the target gene was assessed using the 2^−ΔΔCt^ method.

### 2.8. 16S rRNA Sequencing and Gut Microbiota Analysis

Total microbial DNA from cecal contents was extracted using the QIAamp DNA Stool Kit (Qiagen, Valencia, CA, USA). PCR was used to amplify the bacterial 16S rRNA gene’s V3–V4 region. PCR products were purified using Vazyme VAHTSTM DNA Clean Beads (Vazyme, Shanghai, China), and quantification was performed using a PicoGreen dsDNA Assay Kit (Invitrogen, Carlsbad, CA, USA), which matched our previously published methodology [32]. Sequencing of the 16S rRNA was conducted on the Illumina Novaseq_PE250 sequencing platform, with sequencing services provided by Personal Biotechnology Co., Ltd., Shanghai, China.

### 2.9. Statistical Analysis

The data were analyzed using one-way analysis of variance (ANOVA), followed by Duncan’s multiple comparison test in IBM SPSS Statistics version 22.0 (IBM, Armonk, NY, USA). The linear and quadratic comparisons were applied to identify the dose-effect of *C. nudiflora* aqueous extract in broilers. A Student’s *t*-test and Wilcoxon rank-sum test were conducted for CNEH and CON groups in subsequent experiments. Graphs were generated using GraphPad Prism version 7.0 (GraphPad, San Diego, CA, USA). Statistical significance was defined as *p* < 0.05.

For microbiota profiling, the data gathering and examination were carried out using the Genes Cloud Platform (www.genescloud.cn, accessed on 18 December 2023). To represent the diversity and richness of bacteria, alpha diversity (Chao1, Observed_species, Shannon, and Simpson) was computed. To visualize the complex data, Bray–Curtis dissimilarities were used as the basis for Principal Coordinate Analysis (PCoA). The linear discriminant analysis effect size (LEfSe) method (http://huttenhower.sph.harvard.edu/lefse/, accessed on 20 December 2023) was used to implement the linear discriminant analysis (LDA) distribution histogram. Predictive functional profiling of the bacterial community in cecal content samples of white-feathered broilers was performed using PICRUSt2 (https://github.com/picrust/picrust2, accessed on 22 December 2023) with the KEGG database (https://www.genome.jp/kegg/pathway.html, accessed on 22 December 2023). Spearman correlation was used to investigate the relationship between representative cecal microbiota and different indicators.

## 3. Results

### 3.1. Growth Performance of Broilers

The effects of dietary *C. nudiflora* aqueous extract (CNE) on the growth performance of broilers are shown in Table 1. Dietary CNE supplementation quadratically (*p* < 0.01) increased BW at 21 and 42 days, increased ADG, and decreased F/G on days 1–21 and 1–42. Specifically, the CNEL and CNEM groups noticeably increased the BW on days 21 and 42 compared to the CON group (*p* < 0.05). During days 1~21 and days 1~42, the CNEL and CNEM groups raised ADG (*p* < 0.05) and reduced F/G (*p* < 0.05) compared with the CON group. From 22 to 42 days, no difference in ADG and F/G was observed among dietary treatments (*p* > 0.05). During days 1 to 21, days 22 to 42, and the whole period, no significant differences in ADFI were observed among the four groups (*p* > 0.05).

### 3.2. Immune Organ Indices and Serum Immunoglobulin and Cytokines Level

The results of the immune organ indices and serum immune parameters of broilers are presented in Table 2. Dietary CNE supplementation raised the spleen index (linear, *p* < 0.05), the thymus index (linear, *p* < 0.05), and the levels of serum IgA (quadratic, *p* < 0.01), as well as decreased IL-6 (linear, *p* < 0.01) and TNF-α (linear, *p* < 0.01; quadratic, *p* < 0.05) on day 21. In 42-day-old broilers, the levels of serum IL-6 (linear, *p* < 0.01) and TNF-α (linear, *p* <  0.01) decreased, and the level of serum IL-10 (linear, *p*  <  0.05; quadratic, *p* <  0.05) increased with the addition of CNE. Specifically, the spleen index and thymus index in the CNEM and CNEH groups were notably higher than those of the CON group (*p* < 0.05), while the content of serum IgA and IgM in CNEL group was remarkably lower than that of the CON group (*p* < 0.05). The CNEM group showed a marked increase in the content of serum IgA on day 21 (*p* < 0.05) and IL-10 on day 42 (*p* < 0.05) compared with the CON group, while the spleen index exhibited a noteworthy rise in the CNEH and CNEL groups (*p* < 0.05). No significant changes were noticed on the bursa index or serum IL-10 among four groups of 21-day-old broilers. However, on day 42, there were no variations (*p* > 0.05) observed in the thymus index, bursa index, IgA, or IgM among all groups.

### 3.3. Serum Antioxidant Parameters in Broilers

Table 3 shows the serum antioxidant capacity of broilers fed with CNE. Dietary CNE supplementation increased SOD and T-AOC activity (linear, *p* < 0.01) on days 21 and 42, as well as GSH-Px activity (linear, *p* < 0.01) on day 42, while reducing serum MDA concentrations (linear, *p* < 0.05; quadratic, *p* < 0.05) on days 21 and 42. In comparison to the CON group, the CNEM and CNEH groups showed a noteworthy increase in the activity of GSH-Px and T-AOC (*p* <0.05) on day 42 and SOD (*p* < 0.05) on days 21 and 42. Furthermore, the activity of T-AOC was markedly higher in the CNEH group than that of the CON group on day 21 (*p* < 0.05). Furthermore, the GSH-Px activity of the four groups in 21-day-old broilers did not differ significantly (*p* > 0.05).

### 3.4. Serum Growth Hormone in Broilers

Table 4 shows the effects of CNE on the serum growth hormone of broilers. Supplementing with dietary CNE linearly (*p* < 0.05) and quadratically (*p* < 0.05) raised the level of serum IGF-1 at 21 days, as well as linearly (*p* < 0.01) increasing the level of serum GH at 42 days. In comparison with CON group, the CNEM group markedly raised the level of serum IGF-1 and GH on days 21 and 42, respectively, while the CNEH group increased the level of serum GH on day 42 (*p* < 0.05). Nevertheless, there were no variations in serum GH at 21 days of age or serum IGF-1 at 42 days of age (*p* > 0.05).

### 3.5. Immune-Related Gene Expression in Liver and Jejunum

**Figure 2:**
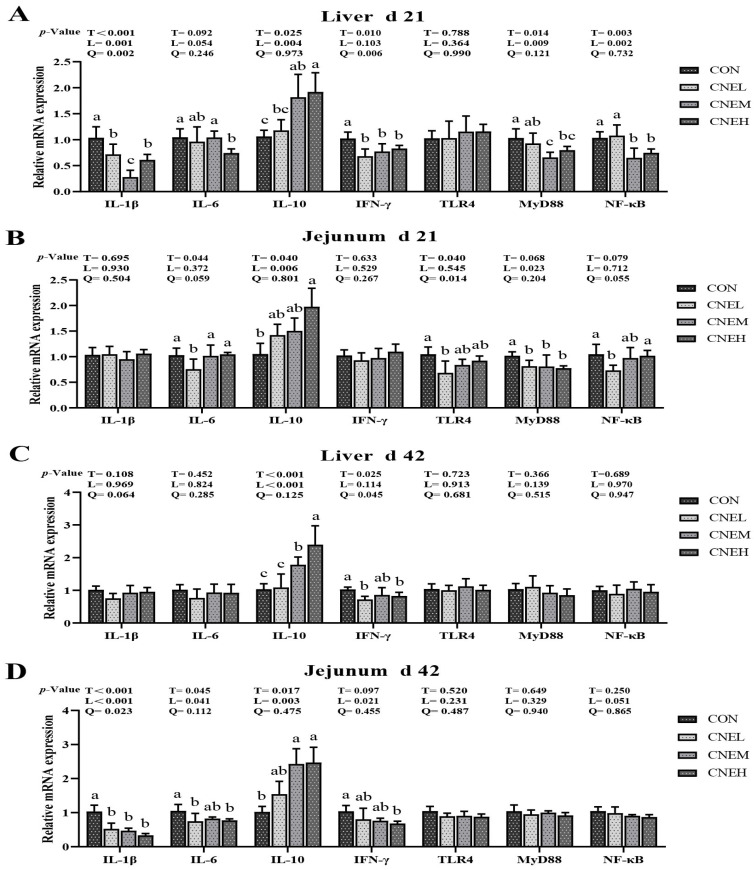
Effects of dietary *C. nudiflora* aqueous extract (CNE) supplementation on the immune-related gene expression of broilers at 21 and 42 days of age. (**A**–**D**) The expression levels of immune-related gene (IL-1β, IL-6, IL-10, IFN-γ, TLR4, MyD88, and NF-κB) in liver and jejunum on day 21 and 42, respectively. IL-1β, interleukin 1 beta; IL-6, interleukin 6; IL-10, interleukin 10; IFN-γ, interferon-gamma; TLR4, Toll-like receptor 4; MyD88, myeloid differentiation factor 88; NF-κB, nuclear factor kappa B. Treatments: CON, control group, basal diet; CNEL, the control diet + 300 mg/kg of CNE; CNEM, the control diet + 500 mg/kg of CNE; CNEH, the control diet + 700 mg/kg of CNE. Values are means ± SD (*n* = 6). T, treatment effect; L and Q, linear and quadratic effect of CNE doses. Means within a column with different superscripts are significantly different (*p* < 0.05).

To investigate the effect of CNE on the expression of immune status in broilers, the relative mRNA expression of immune-related genes in both liver and jejunal mucosa was measured (Figure 2). For the liver of broilers, dietary CNE supplementation raised the mRNA expression of NF-κB (linear, *p* < 0.01) and IL-1β (linear, *p* < 0.01; quadratic, *p* < 0.01) on day 21, as well as IFN-γ (quadratic, *p* < 0.01) on days 21 and 42. The addition of CNE up-regulated the mRNA expression of jejunal IL-1β (linear, *p* < 0.01; quadratic, *p* < 0.05) and IFN-γ (linear, *p* < 0.05) on days 42, as well as jejunal TLR4 (quadratic, *p* < 0.05) on days 21. Regarding the liver and jejunum of broilers, the addition of dietary CNE increased MyD88 (linear, *p* < 0.05) expression on day 21 and up-regulated mRNA expression of IL-10 (linear, *p* < 0.01) on days 21 and 42. Specifically, compared with the CON group, CNEM and CNEH groups significantly up-regulated IL-10 (d 21 and 42) expression and decreased MyD88 and NF-κB (d 21) expression in the liver, while also increasing jejunal IL-10 (d 42) expression (*p*  <  0.05). The CNEH group considerably reduced the relative mRNA expression of IL-6 (d 21) and IFN-γ (d 42) in the liver (*p* < 0.05), while up-regulating jejunal IL-10 (d 21) and down-regulating jejunal IL-6 and IFN-γ (d 42) in comparison to the CON group (*p* < 0.05). Lower expression of IFN-γ (d 42) in the liver and IL-6 (d 21 and 42), TLR4 (d 21), and NF-κB (d 21) in the jejunal was observed when CNEL was added to diets (*p* < 0.05). On day 21, however, there were no appreciable differences in the relative mRNA expressions of IL-1β and IFN-γ in the liver and IL-1β and TLR4 in the jejunal mucosa (*p* > 0.05). The expression of IL-1β and IL-6 in the liver, as well as TLR4, MyD88, and NF-κB in the liver and jejunal mucosa, remained unchanged at 42 days of age (*p* > 0.05).

### 3.6. Antioxidant-Related Gene Expression in Liver and Jejunum

**Figure 3:**
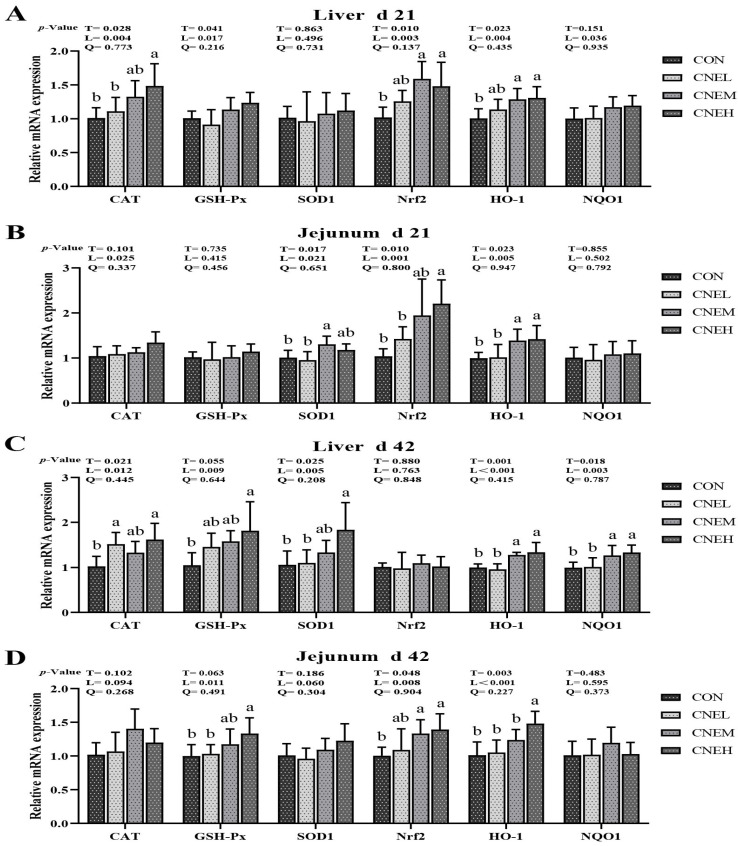
Effects of dietary *C. nudiflora* aqueous extract (CNE) supplementation on the antioxidant-related gene expression of broilers at 21 and 42 days of age. (**A**–**D**) the expression levels of antioxidant-related genes (CAT, GSH-Px, SOD1, Nrf2, HO-1, and NQO1) in liver and jejunum on days 21 and 42, respectively. CAT, catalase; GSH-Px, glutathione peroxidase; SOD1, copper and zinc superoxide dismutase; Nrf2, nuclear factor E2-related factor 2; HO-1, heme oxygenase-1; NQO1, NAD(P)H quinone oxidoreductase 1. Treatments: CON, control group, basal diet; CNEL, the control diet + 300 mg/kg of CNE; CNEM, the control diet + 500 mg/kg of CNE; CNEH, the control diet + 700 mg/kg of CNE. Values are means ± SD (*n* = 6). T, treatment effect; L and Q, linear and quadratic effect of CNE doses. Means within a column with different superscripts are significantly different (*p* < 0.05).

The relative mRNA expression of antioxidant enzymes and the Nrf2 pathway in the liver and jejunum was assessed to examine the impact of CNE on the mRNA expression of antioxidant-related genes in broilers (Figure 3). The mRNA expression of Nrf2 (linear, *p* < 0.05) on day 21, GSH-Px (linear, *p* < 0.05) on day 42, and HO-1 (linear, *p* < 0.05) on days 21 and 42 in the liver and jejunum of the broiler was up-regulated in response to increasing levels of CNE in the diet. As the amount of CNE in the diet increased, there was an up-regulation of mRNA expression in the liver of broilers on day 21 for Nrf2 (linear, *p* < 0.01) and GSH-Px (linear, *p* < 0.05), as well as on day 42 for CAT (linear, *p* < 0.05), SOD1 (linear, *p* < 0.01), and NQO1 (linear, *p* < 0.01). The mRNA expression of jejunal SOD1 (linear, *p* < 0.05) on day 21 and jejunal Nrf2 (linear, *p* < 0.01) on day 42 increased with increasing levels of CNE. On day 21, the CNEM and CNEH groups showed significantly higher Nrf2 expression in the liver and HO-1 expression in the liver and jejunal mucosa than the CON group. At 21 days of age, the CNEH group had remarkably higher mRNA expressions of jejunal Nrf2 and CAT in the liver and jejunal mucosa (*p* < 0.05), while the CNEM group had significantly higher jejunal SOD1 expression than the CON group. However, at 21 days of age, dietary CNE possessed no appreciable impact on the mRNA expression of GSH-Px and NQO1 in the liver and jejunal mucosa and SOD1 in the liver. At 42 days, the CNEM and CNEH groups exhibited higher HO-1 and NQO1 expression in the liver and jejunal Nrf2 expression (*p* < 0.05), while the CNEL and CNEM groups up-regulated CAT expression in the liver and jejunal mucosa, respectively (*p* < 0.05). The mRNA expressions of GSH-Px in the liver and jejunal mucosa, CAT and SOD1 in the liver, and HO-1 in jejunal mucosa were notably (*p* < 0.05) higher in the CNEH group than that of the CON group. At 42 days of age, there was no obvious difference (*p* > 0.05) among diets in the CNE of the mRNA expression of SOD1 and NQO1 in jejunal mucosa and Nrf2 in the liver among the four groups.

### 3.7. Growth-Hormone-Related Factor Gene Expression in Liver and Jejunum

**Figure 4:**
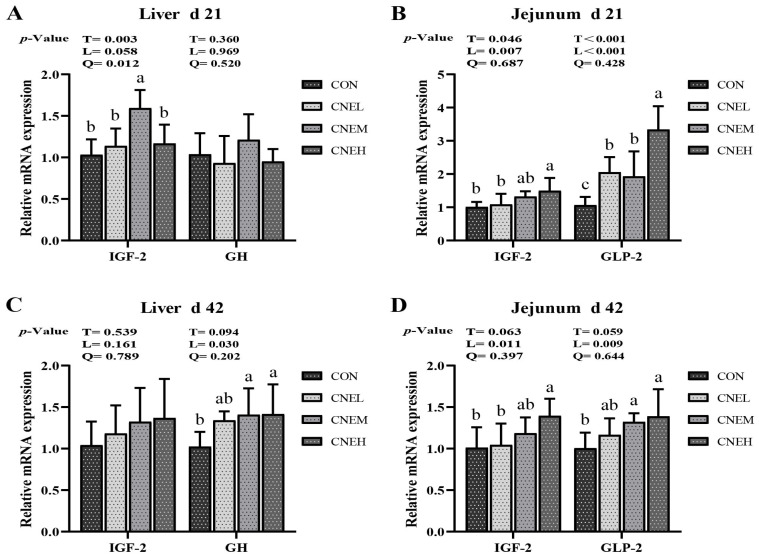
Effects of dietary *C. nudiflora* aqueous extract (CNE) supplementation on the growth-hormone-related factor gene expression of broilers at 21 and 42 days of age. (**A**–**D**) the expression levels of growth-related factor genes (IGF-2, GH, and GLP2) in liver and jejunum on days 21 and 42, respectively. IGF-2, insulin-like growth factor 2; GH, growth hormone; GLP2, glucagon-like peptide 2. Treatments: CON, control group, basal diet; CNEL, the control diet + 300 mg/kg of CNE; CNEM, the control diet + 500 mg/kg of CNE; CNEH, the control diet + 700 mg/kg of CNE. Values are means ± SD (*n* = 6). T, treatment effect; L and Q, linear and quadratic effect of CNE doses. Means within a column with different superscripts are significantly different (*p* < 0.05).

The relative mRNA expression of growth-hormone-related factor genes in the liver and jejunum was evaluated to examine the impacts of CNE on the growth hormone factor in broilers (Figure 4). The increase in dietary CNE supplementation was accompanied by an up-regulation of the mRNA expression levels of GH (linear, *p* < 0.05) at day 42 and IGF-2 (quadratic, *p* < 0.05) at day 21 in the liver. Growing amounts of CNE in the diet on days 21 and 42 led to an up-regulation of the relative mRNA expression of jejunal IGF-2 (linear, *p* < 0.05) and GLP2 (linear, *p* < 0.05). Compared to the CON group, the CNEM group significantly up-regulated IGF-2 expression in the liver, while the CNEH group significantly up-regulated jejunal IGF-2 expression on day 21 and 42. The mRNA expression of GLP-2 in the jejunal mucosa and GH in the liver was significantly higher in the CNEM and CNEH groups at 42 days of age than in the CON group.

### 3.8. Intestinal Morphology and Genes mRNA Levels of Barrier Function and Nutrient Transporter

**Figure 5:**
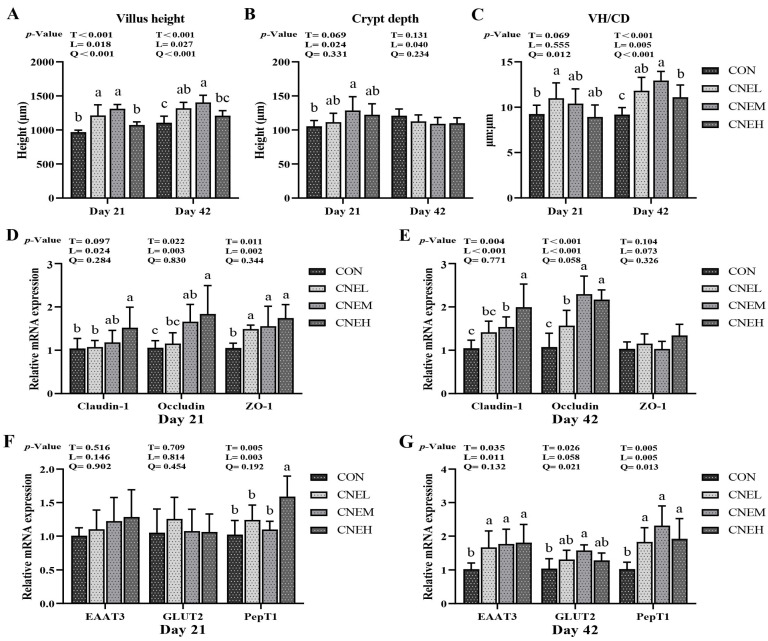
The impact of adding *C. nudiflora* aqueous extract (CNE) to the diet on the expression of nutrient transporter genes, barrier function genes, and jejunal morphology in broilers at 21 and 42 days of age. (**A**–**C**) Jejunal villus height, crypt depth, and VH/CD ratio. (**D**,**E**) The expression levels of tight junction protein (Claudin-1, Occludin, and ZO-1) in jejunum on days 21 and 42, respectively. (**F**,**G**) The expression levels of nutrient transporter genes (EAAT3, GLUT2, and PepT1) in jejunum on day 21 and 42, respectively. ZO-1, glucagon-like peptide 2; EAAT3, excitatory amino acid transporters 3; GLUT2, glucose transporter 2; PepT1, peptide-transporter 1. Treatments: CON, control group, basal diet; CNEL, the control diet + 300 mg/kg of CNE; CNEM, the control diet + 500 mg/kg of CNE; CNEH, the control diet + 700 mg/kg of CNE. Values are means ± SD (*n* = 6). T, treatment effect; L and Q, linear and quadratic effect of CNE doses. Means within a column with different superscripts are significantly different (*p* < 0.05).

The morphology of the jejunal villus of broilers from four groups was well preserved on days 21 and 42 (Figure S1). The jejunum in broilers showed an up-regulation of mRNA expression of crypt depth (linear, *p* < 0.05) on day 21, VH/CD (linear, *p* < 0.05) on day 21, VH/CD (linear, *p* < 0.01; quadratic, *p* < 0.01) on day 42, and villus height (linear, *p* < 0.05; quadratic, *p* < 0.01) on days 21 and 42 in response to rising levels of CNE in the diet. In the jejunum, the villus height in CNEL and CNEM groups was obviously higher than that of the CON group on days 21 and 42 (*p* < 0.05), while the crypt depth in CNEM group and the VH/CD in CNEL group were remarkably higher than that of the CON group on days 21 (*p* < 0.05). Next, we explored the expression of genes associated with tight junctions in the jejunal mucosa of broilers on days 21 and 42 (Figure 5D,E). The mRNA expression of jejunal Claudin-1 and Occludin showed significant increases (linear, *p* < 0.05) on days 21 and 42 with increasing CNE doses, while the jejunal ZO-1 expression also increased (linear, *p*  <  0.01) on days 21. The CNEM and CNEH groups had higher expression of Occludin on day 21 (*p * <  0.05) and Claudin-1 on day 42 (*p*  <  0.05) compared to the CON group. The CNEH group exhibited a significant increase in Claudin-1 expression on day 21 and ZO-1 expression on day 42 in contrast to the CON group. Furthermore, we examined the expression of genes involved in jejunal nutrient transport gene of broiler chickens on days 21 and 42 (Figure 5F,G). Increased dietary CNE supplementation resulted in up-regulation of mRNA expression levels of EAAT3 (linear, *p* < 0.05), GLUT2 (quadratic, *p* < 0.05), and PepT1 (linear, *p* < 0.01; quadratic, *p* < 0.05) at day 42, as well as jejunal PepT1 (linear, *p* < 0.01) at day 21. Compared to the CON group, the CNEH group obviously increased PepT1 expression on day 21 (*p* < 0.05), while CNEM group had a higher GLUT2 expression on day 42 (*p*  <  0.05).

### 3.9. Bacterial Community Diversity and Composition of Cecal Microbiota

**Figure 6:**
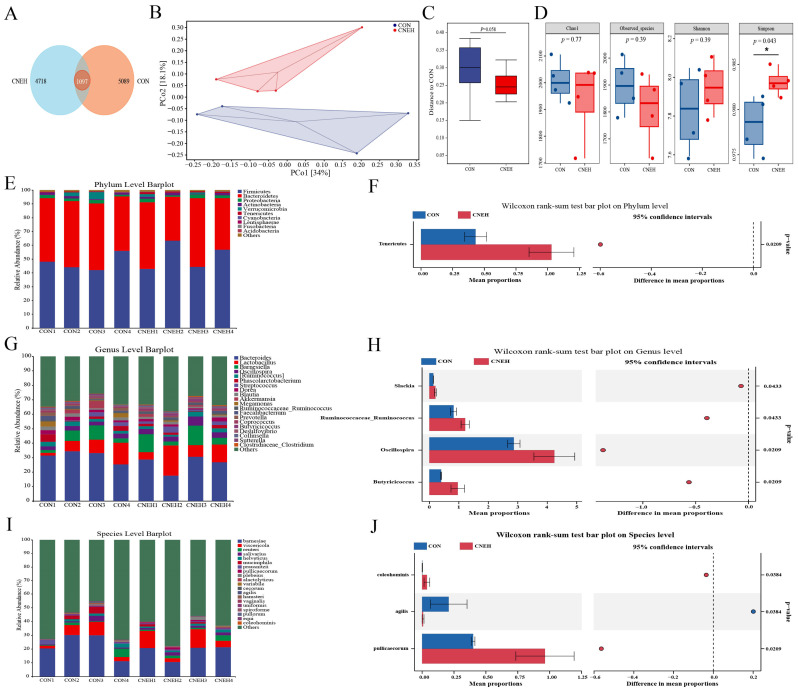
Dietary *C. nudiflora* aqueous extract at 700 mg/kg (CNEH) altered the composition and community diversity of the cecal microbiota at 42 day of age. (**A**) Venn diagram. (**B**,**C**) PCoA analysis and Anosim analysis based on Bray–Curtis distance. (**D**) Alpha-diversity. The bar plots of microbial composition and bacterial taxa show notable phylum-level differences (**E**,**F**), genus (**G**,**H**), and species (**I**,**J**) levels. Treatments: CON, control group, basal diet; CNEH, the control diet + 700 mg/kg of CNE. * indicates a significant difference at *p* < 0.05 (*n* = 4).

**Figure 7:**
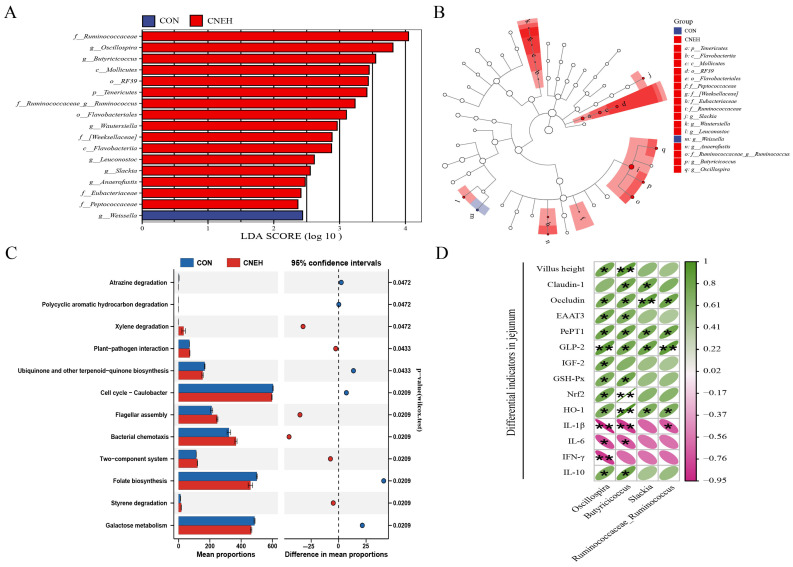
Changes in 42-day-old broiler gut microbiota and predicted function with 700 mg/kg *C. nudiflora* aqueous extract (CNEH) supplementation. (**A**,**B**) Analysis of intestinal microbes using linear discriminant analysis (LDA) effect size (LEfSe) (LDA > 2.0, *p* < 0.05). (**C**) Comparison of predicted pathway abundances among two groups. (**D**) The cecal microbiota composition and differential indicators in jejunum were analyzed using Spearman’s correlation analysis. Treatments: CON, control group, basal diet; CNEH, the control diet + 700 mg/kg of CNE. * indicates a significant difference at *p* < 0.05 and ** indicates *p* < 0.01 (*n* = 4).

The impact of CNEH on gut microbiota was examined using 16S rRNA gene sequencing on fecal samples from the CON and CNEH groups. Following sequencing analysis, each sample yielded an average of 85,463 raw data, and after quality trimming and chimera checking, each sample yielded 56,855 high-quality sequences. The Venn analysis of operational taxonomic units (OTUs) in the cecal contents of 42 d-broilers (Figure 6A) revealed that the CON and CNEH groups contained 5069 and 4718 unique OTUs, respectively, and 1097 shared OTUs. Bray–Curtis distance-based PCoA of the microbial community revealed that the structure of cecal microbial community had changed between CON and CNEH groups, which formed two distinct clusters (Figure 6B,C). Regarding the α-diversity indices, broilers fed with CNEH had higher Simpson indices of cecal microflora than those in the CON group (Figure 6D). However, there was no significant statistical difference in Chao1, observed species, or Shannon indices between these two groups. Next, the analysis also included a comparison of the cecal bacterial compositions of the CON and CNEH groups. More than 90% of the total bacterial phylum in the CON and CNEH groups was represented by the two most prevalent bacterial phyla, *Firmicutes* and *Bacteroidetes* (Figure 6E). The findings of the differential analysis also demonstrated a significant increase in *Tenericutes* abundance by the CNEH group. (Figure 6F). At the genus level (Figure 6G), *Bacteroides* and *Lactobacillus* were the two most abundant genera in the cecal microbiota of broilers, followed by *Barnesiella* and *Oscillospira*. The relative abundances of *Oscillospira*, *Ruminococcaceae_Ruminococcus*, *Butyricicoccus*, and *Slackia* were increased (*p* < 0.05) in the broilers fed with CNEH (Figure 6H). At the species level (Figure 6I,J), an enhanced proportion of *Butyricicoccus_pullicaecorum* and *Lactobacillus_coleohominis*, and reduced *Lactobacillus_agilis* were observed in the CNEH group (*p* < 0.05). Moreover, we employed LEfSe to identify bacterial taxa that significantly differentiated between these two groups (Figure 7A,B), and the result revealed that 16 bacterial biomarkers were enriched (LDA threshold  >  2.5) in the caecum of the CNEH group, including *f_Ruminococcaceae* (family), *g_Oscillospira*, and *g_Butyricicoccus* (LDA score > 3.5, *p* < 0.05).

### 3.10. Functional Prediction of Cecal Microbes

The changes in the metabolic pathways of the predictive functional profiles from the cecal microbiota are shown in Figure 7C. The dietary inclusion of CNEH led to a significant increase in the microbial gene abundance related to xenobiotics biodegradation and metabolism (xylene degradation and styrene degradation) across all three levels of the KEGG pathways compared to the CON group. Conversely, CNEH group resulted in a decrease (*p* < 0.05) in the gene abundance associated with carbohydrate metabolism (galactose metabolism), metabolism of cofactors and vitamins (folate biosynthesis and ubiquinone and other terpenoid-quinone biosynthesis), and xenobiotics biodegradation and metabolism (atrazine degradation and polycyclic aromatic hydrocarbon degradation).

### 3.11. Correlation Analysis between Cecal Microbes and Different Indicators

The conceivable interconnections between the main cecal microbiota genus and all significantly different jejunal parameters were examined in this study using a Spearman correlation analysis (Figure 7D). These parameters included jejunal villi (Villus height), barrier function (Claudin-1 and Occludin), nutrient transporters (EAAT3 and PePT1), growth factors (GLP-2 and IGF-2), antioxidant parameters (GSH-Px, Nrf2, and HO-1), and immune parameters (IL-1β, IL-6, IFN-γ, and IL-10). In this study, *Oscillospira* and *Butyricicoccus* demonstrated positive correlations with villus height and the mRNA expression of Occludin, EAAT3, PePT1, GLP2, GSH-Px, Nrf2, HO-1, and IL-10 in the jejunum (*p* < 0.05), while exhibiting negative correlations with IL-1β and IL-6 expression in the jejunum (*p* < 0.05). The abundance of *Oscillospira* showed positive correlations with IGF-2 (*p* < 0.05) and negative correlations with IFN-γ expression (*p* < 0.01), whereas the abundance of *Butyricicoccus* was positively correlated with Claudin-1 expression and negatively correlated with GLP-2 and TLP2, respectively. The abundance of *Slackia* and *Ruminococcaceae_Ruminococcus* had a positive correlation with the mRNA expression of Occludin, PePT1, GLP2, and HO-1 in the jejunum (*p* < 0.05). Additionally, *Slackia* exhibited a positive correlation with Claudin-1 (*p* < 0.05) expression, whereas *Ruminococcaceae_Ruminococcus* demonstrated a negative association with the mRNA expression of IL-1β (*p* < 0.05) in the jejunum, respectively.

## 4. Discussion

The use of phytogenic feed additives and extracts to enhance broiler health had become increasingly popular over time, as evidenced by numerous studies [33]. Research has indicated that the extract of *C. nudiflora* includes polyphenols, essential oils, terpenoids, flavonoids, and other active ingredients [15]. These medicinal plants have anti-inflammatory and antioxidant properties that may be related to bioactive substances (terpenoids, flavonoids, and polyphenols) that improve the health and productivity of broiler chickens [34,35]. In the present investigation, broilers fed diets supplemented with CNE at 300 and 500 mg/kg improved the growth performance of broilers by increasing the average daily gain (ADG) and decreasing the feed-to-gain ratio (F/G) during the entire period (days 1 to 42). Throughout the entire period and in the grower and finisher phases, birds fed 1% of *Lippia citriodora* leaf powder containing verbascoside had a positive impact on feed intake, feed conversion ratio, and average daily gain [36]. Some studies have found that the ADG and ADFI in broilers increased when luteolin was added to deoxynivalenol (DON)-containing diets [37]. The better overall performance of broilers fed CNE might be due to the presence of luteolin and verbascoside from *Callicarpa nudiflora*, which might improve nutrient absorption.

According to Kuhn et al. [38], GH could promote the development and differentiation of muscle and bone cells. Additionally, many of GH’s growth-promoting effects in poultry were elicited by stimulating IGF-1 efflux via the liver or activating cellular GH receptors, as suggested by Anh et al. [39]. In this study, dietary supplementation with 500 mg/kg CNE up-regulated the expression of GLP-2 in the jejunum and GH in the liver, as well as the serum GH and IGF-1 level. Based on previous research, broiler IGF-I mRNA expression was up-regulated, and serum GH levels were elevated after genistein (40 mg/kg) treatment [40]. A study in piglets showed that rutin, as a cheap polyphenolic flavonoid with various biological activities, could increase serum GH content when added to the feed at 500 mg/kg [41]. Therefore, dietary CNE had the effect of increasing serum GH and IGF-1 content, which might further explain the improvement of the growth performance of broilers. However, the improvement of the active components of CNE in growth hormone needs further study.

Antioxidant enzymes such as glutathione peroxidase (GSH-Px), catalase (CAT), and superoxide dismutase (SOD) were recognized for their significant role in scavenging free radicals, thereby contributing to antioxidant defense mechanisms [42]. The total antioxidant status of both enzyme and non-enzyme defense systems was assessed by T-AOC, while reactive oxygen species (ROS) generation leading to lipid peroxidation resulted in the production of MDA, which served as an end product and a proxy for assessing the extent of cell damage [43]. Our findings revealed that CNE could improve broiler antioxidant capacity by increasing serum T-AOC enzyme activity (day 42) and decreasing serum MDA content, whereas CNE at 500 and 700 mg/kg could increase serum SOD and GSH-Px enzyme activities. Similar to other studies, it was discovered that diets supplemented with 1000 mg/kg *Artemisia annua* L. leaf extract containing the phenolic compounds and flavonoids increased SOD, CAT, and GPx activities while decreasing MDA concentrations in serum [44]. Luteolin had the capacity to lessen NH4CL-induced peroxidation, as evidenced by the fact that adding luteolin to the experimental setup reduced the content of MDA within the cell while increasing the activity of SOD and GSH-Px [45]. While verbascoside has been shown to have relatively modest direct reactive oxygen species scavenging activities, its metabolites have been shown to significantly increase the activities of major antioxidant enzymes (CAT, GSH-Px, and glutathione reductase) while also suppressing MDA related to inflammation and pro-oxidants in the circulating neutrophils, erythrocytes, and lymphocytes of rats after consuming lemon verbena extract [46,47]. Furthermore, in the present study, we observed the level (700 mg/kg) of CNE up-regulated CAT expression (d 21 and 42) and GSH-px expression (d 42) in the liver and jejunal mucosa. In particular, the CNE at 500 and 700 mg/kg up-regulated HO-1 expression in the liver and HO-1/Nrf2 expression in jejunal mucosa. According to a previous study, the butanolic extract (BE) from *Nelumbo nucifera* leaves, which had a high level of total phenol and flavonoid, activates HO-1/Nrf2 and antioxidant enzymes to prevent liver damage caused by hydrogen peroxide [48]. The results of bioactive substance analysis show that CNE contains polyphenols and flavonoids and has antioxidant effects. Therefore, the antioxidant components in CNE might contribute to the improvements in antioxidant status observed in broilers from the CNE groups; however, the exact underlying mechanism requires further investigation.

The thymus, spleen, and bursal indices are crucial indicators for evaluating the health of chickens’ immune systems [49]. The ontogenetic development of adaptive immunity depends on the thymus and bursa of Fabricius, which are two key lymphoid structures found exclusively in chickens [50]. In the current study, broilers in the CNE at 500 and 700 mg/kg had a higher spleen index and thymus index on day 21. Additionally, the major serum immune indices are IgA, IgM, and IgG, which indicate the animal body’s degree of immunity and, in turn, the body’s capacity to fend off pathogenic microbes [51]. In this study, the concentrations of serum TNF-α and IL-6 were found to drop when CNE was added to the basal diet. Meanwhile, dietary supplementation with 300 mg/kg CNE increased the levels of serum IgA and IgM. Similarly, Zhang et al. [52] found that adding 200 mg/kg of *Epimedium* Isopentenyl flavonoids to the baseline broiler diet resulted in elevated blood levels of IgA and IgG, accompanied by reduced levels of TNF-α, IL-6, and pro-inflammatory protein IL-1β. NF-κB, considered a crucial downstream mediator in the TLR/MyD88/NF-κB signaling cascade, played a significant role in this process [53], where it was essential for coordinating the expression of adhesion molecules, inflammatory factors, and critical cytokines in vivo [53]. Consistent with these facts, we observed in the present study that supplementation with 500 and 700 mg/kg CNE induced immune activation and regulation via the decreased gene expression of IL-1β, IFN-γ, MyD88, and NF-κB in the liver and up-regulated IL-10 expression. Monocytes and macrophages released proinflammatory mediators such as TNF-α, IL-1β, and IL-6; however, the cytokine IL-10 worked to counteract this process by targeting these cells, resulting in a decrease in the release of these proinflammatory mediators [54]. Verbascoside at 20 mg/kg doses prevented the development of osteoarthritis in rats by lowering proinflammatory interleukin (IL-1β, IL-6) and tumor necrosis factor (TNF)-α [55]. The protective and curative effects of verbascoside from plant cell cultures of *Syringa vulgaris* L. have been confirmed by reduced NFκB activation, which has attenuated NFκB-dependent cellular events, such as adhesion factor expression, inducible nitric oxide synthase and poly (ADP ribose) up-regulation, and metalloproteinase (MMP2 and MMP9) activation [56]. Wang et al. [57] extracted several diterpenoids with potent anti-inflammatory properties from the leaves of *Callicarpa nudiflora*. Therefore, it is conceivable that the active components of CNE act as immunostimulants, triggering immune cells and potentially leading to an increase in the anti-inflammatory cytokine IL-10, which maintains homeostasis by suppressing the inflammatory response. However, more research is needed into the specific mechanisms.

The length of the villi and the depth of the crypts show the effective area and function of intestinal digestion and nutritional absorption, and their ratio accurately depicts the small intestine’s functional state [58]. In this research, broilers fed diets supplemented with CNE at 300 and 500 mg/kg increased the VH/CD ratio and villus height in the jejunum. Zhong et al. [59] discovered that phenolic compounds were linked to the capacity of 500 mg/kg *Ilicis chinensis* folium extract (ICFE) to alter intestinal morphology, which enhanced their capacity to take in nutrients from a regular diet. Tight junction proteins serve as the gut’s physical barrier, regulating its permeability, which is crucial for protecting the gastrointestinal tract [60]. Within this current study, adding CNE to broiler diets enhanced jejunal barrier function by up-regulating ZO-1 and Occludin expression. Specifically, CNE at 500 and 700 mg/kg increased the mRNA expression of Claudin-1 and Occludin. A study found that supplementation with Eleutheroside B (a phenylpropanoid glycoside) led to higher levels of intestinal tight junction protein expression, both mRNA and protein (Claudin-3, Occludin, and ZO-1) in IPEC-J2 cells [61]. The gut played a crucial role in the process of the digestion and absorption of nutrients, thus significantly impacting the productivity and well-being of animals. In particular, the nutrient transporters located in the small intestine’s apical membrane were essential for transferring nutrients into enterocytes [62]. This study discovered that diets supplemented with 500 mg/kg CNE up-regulated mRNA expression of nutrient transporters (PepT1, EAAT3, and GLUT2) in the jejunum. Similarly, it was widely recognized that feed conversion ratio (FCR), weight gain, and body weight of broilers were all impacted by variations in the relative abundance of intestinal transporters, including PepT1, GLUT2, and EAAT3 [63]. According to these findings, dietary CNE supplementation improves the function of the jejunal barrier and aids in the absorption of nutrients, both of which might contribute to the improvement of intestinal health. However, the effects of CNE on nutrient digestion need to be further investigated.

The microbial population in the animal’s gut is essential for maintaining intestinal health, physiological function and regulating the host immune system [21]. In the analysis of microflora, the PCA analysis comparing the β-diversity of the cecal microbial community showed distinct and well-differentiated microbiota between CNEH and CON groups. The addition of CNEH to diets increased the Simpson index of cecum. This suggests that CNEH changed cecal microbial diversity. Interestingly, the abundance of *Oscillospira*, *Ruminococcaceae_Ruminococcus*, *Butyricicoccus*, and *Slackia* in the CNEH group was higher. In addition to belonging to the *Ruminococcaceae* family, the *Oscillospira* genus is known for its production of butyrate, a short-chain fatty acid [64]. The *Ruminococcaceae* family (*Firmicutes*) consists of bacteria known for producing acetate, lactate, and butyrate [65], which are crucial for maintaining epithelial tissue health and limiting the growth of pathogenic bacteria [66]. Additionally, Geirnaert et al. [67] identified *Butyricicoccus pullicaecorum* as a naturally occurring probiotic that produces butyrate and exhibits innate resistance to conditions in the stomach and small intestine. The relative abundance of *Oscillospira*, *Ruminococcaceae_Ruminococcus*, and *Butyricicoccus*, which are producers of SCFAs, increases in the CNEH group. In line with the results of this investigation, flavonoids have been demonstrated to have prebiotic properties by inducing the microbial synthesis of lactic acid and short-chain fatty acids [68]. Changes in the diversity and composition of the intestinal microbiota in broilers have been observed, and these shifts might have had an impact on the growth, metabolism, and host health of gut microbes. The PICRUSt2 results revealed six metabolic function pathways in the cecal flora of the CNEH group, including xenobiotic biodegradation metabolism and carbohydrate metabolism. These results were similar to those of Zhu et al. [69], who reported that the cecal microbiota metabolic pathway of the plant extracts group (400 mg/kg) in broilers on the 14th day was associated with amino-acid-related enzymes, xenobiotics biodegradation metabolism, the D-Arginine and D-ornithine metabolism, and carbohydrate metabolism. The performance gains seen in the chickens fed CNE could have been brought on by similar changes in intestinal microbial function. Consequently, CNE supplementation could promote the development and proliferation of beneficial gut bacteria, and the metabolites produced by these bacteria might contribute to the improvement of gut health. Thus, the growth and reproduction of gut bacteria may be encouraged by the supplementation of CNE, and the metabolites generated by these bacteria may help to improve gut health.

The gut microbiota serve as a critical immune system barrier, influencing the host’s immunological and metabolic systems and strongly influencing overall host health [70]. Spearman’s correlation analysis revealed that *Oscillospira* and *Butyricicoccus* were positively correlated with intestinal integrity, nutrient transporters, growth factors, antioxidant parameters, and immune parameters. The abundance of *Ruminococcus* had a positive correlation with intestinal barrier function, nutrient transporters, and antioxidant parameters in this study. *Oscillospira*, another butyrate-producing bacteria, has the potential to suppress the expression of genes responsible for pro-inflammatory cytokines, thus helping to prevent inflammation in humans [64]. Butyrate possesses various advantageous qualities that are essential for maintaining intestinal well-being, and *Butyricicoccus pullicaecorum*, which produces butyrate, has probiotic potential [67]. The *Ruminococcaceae* groups, recognized for their production of short-chain fatty acids (SCFAs), indirectly contribute to butyrate synthesis by generating acetate, lactate, and succinate for use in interconversion processes [71]. SCFAs, which are produced through bacterial fermentation, play crucial roles in maintaining gut integrity, regulating the immune system, reducing inflammation, and modulating epithelial gene expression [72]. Hence, dietary supplementation with CNE in broilers demonstrated improved growth performance and a range of health benefits (such as enhanced growth hormone, immune system and antioxidant function, intestinal barrier function, and nutrient transporter). These beneficial effects might be related to the regulation of intestinal microbiota by CNE, and it was speculated that CNE might be a potential substitute for APGs in poultry feed additives.

## 5. Conclusions

The findings of our study suggested that the addition of CNE to broilers’ diets might improve their intestinal health and growth performance. These beneficial effects were related to the enhancement of antioxidant function, immunity, growth hormone, intestinal function, and the regulation of cecal microbiota in broilers by CNE. Particularly, the dietary addition of 500 mg/kg of *Callicarpa nudiflora* aqueous extract used in this study was a valuable feed additive for broilers. The addition of CNE to the diet could promote the growth and health maintenance of broilers, but the exact functional mechanism of CNE on broilers remains to be further studied.

## Figures and Tables

**Table 1:** Growth performance in 21-day and 42-day-old broilers as affected by dietary *C. nudiflora* aqueous extract (CNE) supplementation.

Items	Treatments				SEM	*p*-Value		
	CON	CNEL	CNEM	CNEH		T	L	Q
BW								
21 day (g)	796.9 ^b^	820.9 ^a^	816.1 ^a^	806.8 ^ab^	2.99	0.013	0.272	0.003
42 day (g)	2293.3 ^b^	2360.4 ^a^	2345.4 ^a^	2316.7 ^ab^	8.85	0.026	0.423	0.005
1 to 21 day								
ADG (g)	36.28 ^b^	37.45 ^a^	37.23 ^a^	36.77 ^ab^	0.14	0.009	0.247	0.002
ADFI (g)	54.05	53.35	52.11	52.99	0.28	0.098	0.073	0.145
F/G	1.49 ^a^	1.42 ^b^	1.40 ^b^	1.45 ^b^	0.01	0.002	0.015	0.001
22 to 42 day								
ADG (g)	71.26	73.31	72.78	71.90	0.39	0.260	0.688	0.068
ADFI (g)	127.81	129.71	127.96	127.88	0.49	0.492	0.733	0.334
F/G	1.79	1.77	1.76	1.77	0.09	0.431	0.391	0.168
1 to 42 day								
ADG (g)	53.77 ^b^	55.38 ^a^	55.01 ^a^	54.33 ^ab^	0.21	0.024	0.425	0.005
ADFI (g)	90.93	91.53	90.04	90.44	0.28	0.263	0.231	0.852
F/G	1.69 ^a^	1.65 ^b^	1.64 ^b^	1.66 ^ab^	0.01	0.019	0.060	0.008

BW, body weight; ADG, average daily gain; ADFI, average daily feed intake; F/G, feed-to-gain ratio. Treatments: CON, control group, basal diet; CNEL, the control diet + 300 mg/kg of CNE; CNEM, the control diet + 500 mg/kg of CNE; CNEH, the control diet + 700 mg/kg of CNE. Variation in the data was expressed as pooled SEM (*n* = 6). T, treatment effect; L and Q, linear and quadratic effect of CNE doses. ^a,b^ Means within a row with no common superscript differ significantly (*p* < 0.05).

**Table 2:** Immune organ indices and serum immune parameters in 21-day and 42-day-old broilers as affected by dietary *C. nudiflora* aqueous extract (CNE) supplementation.

Items	Treatments				SEM	*p*-Value		
	CON	CNEL	CNEM	CNEH		T	L	Q
21 day								
Spleen index (%)	0.075 ^b^	0.088 ^ab^	0.091 ^a^	0.093 ^a^	0.003	0.043	0.010	0.269
Thymus index (%)	0.227 ^b^	0.256 ^ab^	0.247 ^a^	0.296 ^a^	0.009	0.036	0.010	0.523
Bursa index (%)	0.261	0.291	0.267	0.282	0.011	0.774	0.714	0.749
IgA (μg/mL)	321.3 ^c^	380.7 ^a^	359.5 ^ab^	328.7 ^bc^	7.36	0.006	0.988	0.001
IgM (μg/mL)	843.0 ^b^	930.7 ^a^	869.0 ^ab^	897.2 ^ab^	12.66	0.047	0.259	0.163
IL-6 (pg/mL)	35.8 ^a^	33.3 ^ab^	32.3 ^b^	32.0 ^b^	0.51	0.020	0.004	0.218
IL-10 (pg/mL)	103.8	99.1	101.6	101.3	0.94	0.385	0.550	0.257
TNF-α (pg/mL)	108.0 ^a^	105.8 ^a^	106.2 ^a^	97.1 ^b^	1.12	<0.001	<0.001	0.033
42 day								
Spleen index (%)	0.085 ^b^	0.120 ^a^	0.099 ^ab^	0.116 ^a^	0.005	0.033	0.067	0.306
Thymus index (%)	0.096	0.100	0.108	0.090	0.004	0.576	0.578	0.507
Bursa index (%)	0.146	0.178	0.157	0.167	0.008	0.560	0.834	0.238
IgA (μg/mL)	343.9	359.6	366.7	344.7	4.4	0.189	0.807	0.039
IgM (μg/mL)	829.8	860.8	836.7	848.5	8.6	0.369	0.150	0.400
IL-6 (pg/mL)	38.7 ^a^	32.9 ^b^	34.1 ^b^	30.7 ^b^	0.83	0.001	<0.001	0.325
IL-10 (pg/mL)	87.0 ^b^	90.8 ^ab^	92.4 ^a^	90.6 ^ab^	0.72	0.036	0.035	0.035
TNF-α (pg/mL)	100.5 ^a^	94.8 ^b^	94.2 ^b^	86.8 ^c^	1.17	<0.001	<0.001	0.482

IgA, immunoglobulin A; IgM, immunoglobulin M; IL-6, interleukin 6; IL-10, interleukin 10; TNF-α, tumor necrosis factor-alpha. Treatments: CON, control group, basal diet; CNEL, the control diet + 300 mg/kg of CNE; CNEM, the control diet + 500 mg/kg of CNE; CNEH, the control diet + 700 mg/kg of CNE. Variation in the data was expressed as pooled SEM (*n* = 6). T, treatment effect; L and Q, linear and quadratic effect of CNE doses. ^a–c^ Means within a row with no common superscript differ significantly (*p* < 0.05).

**Table 3:** Antioxidant capacity of serum in 21-day and 42-day-old broilers as affected by dietary *C. nudiflora* aqueous extract (CNE) supplementation.

Items	Treatments				SEM	*p*-Value		
	CON	CNEL	CNEM	CNEH		T	L	Q
21 day								
SOD (U/mL)	186.6 ^b^	195.8 ^b^	258.3 ^a^	241.7 ^a^	7.92	<0.001	<0.001	0.226
GSH-Px (μmol/L)	43.4	47.1	43.8	45.5	1.26	0.733	0.792	0.693
T-AOC (mmol/L)	0.51 ^b^	0.55 ^b^	0.57 ^ab^	0.64 ^a^	0.02	0.012	0.001	0.651
MDA (nmol/mL)	9.8 ^a^	5.9 ^b^	6.9 ^b^	6.6 ^b^	0.50	0.016	0.029	0.042
42 day								
SOD (U/mL)	202.5 ^c^	213.7 ^bc^	239.5 ^ab^	248.3 ^a^	5.94	0.010	0.001	0.907
GSH-Px (μmol/L)	31.6 ^b^	32.8 ^b^	41.5 ^a^	42.6 ^a^	1.52	0.005	0.001	0.973
T-AOC (mmol/L)	0.51 ^b^	0.59 ^a^	0.61 ^a^	0.62 ^a^	0.01	0.002	0.001	0.059
MDA (nmol/mL)	11.2 ^a^	6.2 ^b^	8.0 ^b^	6.8 ^b^	0.53	0.001	0.003	0.022

SOD, superoxide dismutase; GSH-Px, glutathione peroxidase; T-AOC, total antioxidant capacity; CAT, catalase; MDA, malonaldehyde. Treatments: CON, control group, basal diet; CNEL, the control diet + 300 mg/kg of CNE; CNEM, the control diet + 500 mg/kg of CNE; CNEH, the control diet + 700 mg/kg of CNE. Variation in the data was expressed as pooled SEM (*n* = 6). T, treatment effect; L and Q, linear and quadratic effect of CNE doses. ^a–c^ Means within a row with no common superscript differ significantly (*p* < 0.05).

**Table 4:** Growth hormone of serum in 21-day and 42-day-old broilers as affected by dietary *C. nudiflora* aqueous extract (CNE) supplementation.

Items	Treatments				SEM	*p*-Value		
	CON	CNEL	CNEM	CNEH		T	L	Q
21 day								
GH (ng/mL)	18.7	18.3	19.4	18.9	0.21	0.267	0.194	0.831
IGF-1 (ng/mL)	154.2 ^b^	158.0 ^b^	175.9 ^a^	161.2 ^b^	2.43	0.003	0.029	0.021
42 day								
GH (ng/mL)	15.4 ^c^	16.5 ^bc^	17.5 ^ab^	18.4 ^a^	0.30	<0.001	<0.001	0.905
IGF-1 (ng/mL)	165.6	159.8	170.3	169.1	3.24	0.691	0.487	0.733

GH, growth hormone; IGF-1, insulin-like growth factor 1. Treatments: CON, control group, basal diet; CNEL, the control diet + 300 mg/kg of CNE; CNEM, the control diet + 500 mg/kg of CNE; CNEH, the control diet + 700 mg/kg of CNE. Variation in the data was expressed as pooled SEM (*n* = 6). T, treatment effect; L and Q, linear and quadratic effect of CNE doses. ^a–c^ Means within a row with no common superscript differ significantly (*p* < 0.05).

## Data Availability

The names of the repository/repositories and accession number can be found below: https://www.ncbi.nlm.nih.gov/sra/PRJNA1053503, accessed on 16 December 2022.

## Supplementary Materials

The following supporting information can be downloaded at: https://www.mdpi.com/article/10.3390/antiox13050572/s1, Figure S1: The jejunal tissues of broilers were stained with H&E; Table S1: The gradient elution procedure of HPLC; Table S2: The commercial kits information; Table S3: The ingredient and nutrient composition of the basal diet; Table S4: Primers used for quantitative PCR.
