# Supplementary material for: Effects of Dietary Callicarpa nudiflora Aqueous Extract Supplementation on Growth Performance, Growth Hormone, Antioxidant and Immune Function, and Intestinal Health of Broilers
Source: Antioxidants (Basel). 2024 May 6;13(5):572. doi: 10.3390/antiox13050572 (PMC11117905; doi:10.3390/antiox13050572)
Supplement: Supplementary file 1 [file antioxidants-13-00572-s001.zip › antioxidants-2955685-supplementary.pdf]

Supplementary information

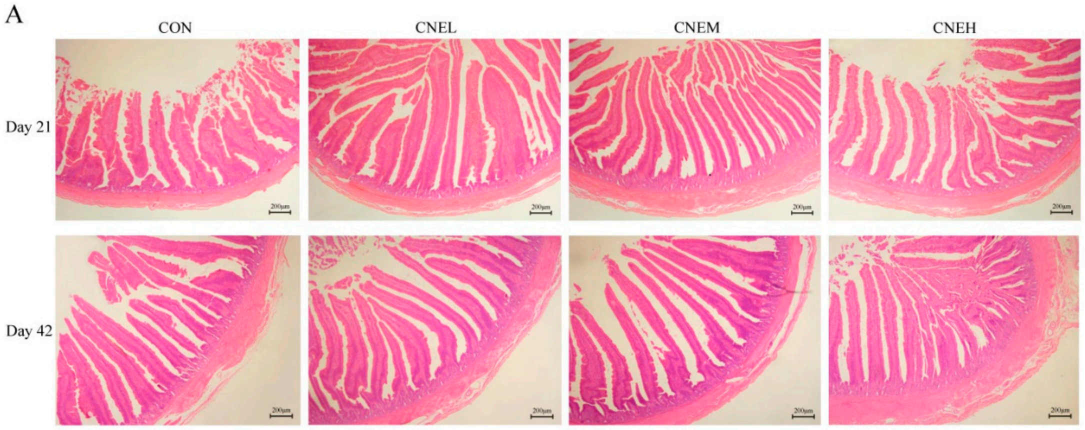

Figure S1: The jejunal tissues of broilers were stained with H&E. Scale bar: 200 μm. (A) Microvillus morphology of the jejunum in 21- and 42-day-old broilers. CON, control group, basal diet; CNEL, the control diet + 300 mg/kg of CNE; CNEM, the control diet + 500 mg/kg of CNE; CNEH, the control diet + 700 mg/kg of CNE.

Table S1: The gradient elution procedure of HPLC.

Time (min)	mobile phase: A (%)	mobile phase: B (%)
0~10	40~50	60~50
10~15	50~60	50~40
15~25	60~70	40~30

Table S2: The commercial kits information.

Items	Name of kits	Cat. No.
Phenol	Plant total phenol test kit	A143-1-1
Flavonoid	Plant flavonoids test kit	A142-1-1
GH	Chicken growth hormone (GH) ELISA Kit	mlC60198-1
IGF-1	Chicken insulin-like growth factor 1 (IGF-1) ELISA kit	mlC60241-1
IgA	Chicken immunoglobulin A (IgA) ELISA kit	mlC60450-1
IgM	Chicken immunoglobulin M (IgM) ELISA kit	mlC60244-1
IL-6	Chicken interleukin-6 (IL-6) ELISA kit	mlC60293-1
IL-10	Chicken interleukin-10 (IL-10) ELISA kit	mlC60245-1
TNF-α	Chicken tumor necrosis factor-α (TNF-α) ELISA kit	mlC60499-1
MDA	Malondialdehyde (MDA) assay kit (TBA method)	A003-1-2
GSH-Px	Glutathione peroxidase (GSH-PX) assay kit (Colorimetric method)	A005-1-2
SOD	Superoxide Dismutase (SOD) assay kit (Hydroxylamine method)	A001-2-2
T-AOC	Total antioxidant capacity (T-AOC) assay kit (ABTS method)	A015-2-1

GH, growth hormone; IGF-1, insulin-like growth factor 1; IgA, immunoglobulin A; IgM, immunoglobulin M; IL-6, interleukin-6; IL-10, interleukin-10; TNF-α, tumor necrosis factor-α; MDA, malondialdehyde; GSH-Px, glutathione peroxidase; SOD, superoxide dismutase; T-AOC, total antioxidant capacity.

Table S3: The ingredient and nutrient composition of the basal diet.

Component	Days 1~21	Days 22~42
Ingredient (%)		
Corn	55.21	62.21
Soybean meal	36.40	29.20
soybean oil	4.70	4.90
Limestone	1.52	1.60
CaHPO4	1.00	1.00
L-Lysine	0.35	0.30
Methionine	0.16	0.13
L-Threonine	0.06	0.06
Salt	0.30	0.30
Premix 1	0.30	0.30
Total	100.00	100.00
Nutrient levels 2		
Metabolic energy, MJ/kg	12.72	13.02
Curde protein, %	20.65	18.28
Lysine, %	1.27	1.09
Methionine, %	0.47	0.41
Calcium, %	0.90	0.91
Available P, %	0.54	0.52

1 Premix is supplied per kg of diet: vitamin A 12000 IU, vitamin D 32500 IU, vitamin E 20.0 mg, vitamin K3 3.0 mg, vitamin B1 3.0 mg, vitamin B2 8.0 mg, vitamin B6 7.0 mg, vitamin B12 0.03 mg, pantothenic acid 20.0 mg, niacin 50.0 mg, biotin 0.1 mg, folic acid 1.5 mg, Fe 45 mg, Cu 17.5 mg, I 1.5 mg, Zn 105 mg, Mn 124 mg, Se 15 mg.

2 Nutrient level is calculated value.

Table S4: Primers used for quantitative PCR.

Gene	Primer sequence (5′→3′)	Accession number
β-actin	Forward: GAGAAATTGTGCGTGACATCA<br>Reverse: ACCTCTGTCATCTCTCCACA	L08165.1
IL-1β	Forward: CAGCCTCAGCGAAGAGACCTT<br>Reverse: ACTGTGGTGTGCTCAGAATCC	NM_204524.2
IL-6	Forward: AAATCCCTCCTCGCCAATCT<br>Reverse: CCCTCACGGTCTTCTCCATAAA	HM179640
IL-10	Forward: CGCTGTCACCGCTTCTTCA<br>Reverse: TCCCGTTCTCATCCATCTTCTC	AJ621614
IFN-γ	Forward: AGCCGCACATCAAACACATA<br>Reverse: CGCTGGATTCTCAAGTCGTT	NM_205149.1
TLR4	Forward: AGGCACCTGAGCTTTTCCTC<br>Reverse: TACCAACGTGAGGTTGAGCC	NM_001030693.1
Myd88	Forward: ATCCGGACACTAGAGGGAGG<br>Reverse: GGCAGAGCTCAGTGTCCATT	NM_001030962.1
NF-κB	Forward: GTGTGAAGAAACGGGAACTG<br>Reverse: GGCACGGTTGTCATAGATGG	NM_205129
CAT	Forward: GGTTCGGTGGGGTTGTCTTT<br>Reverse: CACCAGTGGTCAAGGCATCT	NM_001031215.2
GSH-Px	Forward: GACCAACCCGCAGTACATCA<br>Reverse: GAGGTGCGGGCTTTCCTTTA	NM_001277853.2
SOD1	Forward: ATTACCGGCTTGTCTGATGG<br>Reverse: CCTCCCTTTGCAGTCACATT	NM_205064.155
Nrf2	Forward: CGCTTTCTTCAGGGGTAGCA<br>Reverse: AGTTCGGTGCAGAAGAGGTG	NM_205117.1
HO-1	Forward: ACGAGTTCAAGCTGGTCACG<br>Reverse: GGATGCTTCTTGCCAACGAC	NM_205344.1
NQO1	Forward: GGCAATGGCAGCAGCAG<br>Reverse: AAGCACTCGGGGTTCTTGAG	NM_001277621.1
IGF-2	Forward: CCTTCCTGGCCTATGCGTTG<br>Reverse: TCACAGCTCCGAAAGCAGCA	NM_001030342
GH	Forward: TACGGCCTGCTGTCCTGCTT<br>Reverse: TGTTTTTGGTGACGGGGAGG	NM_204359
GLP-2	Forward: AAGCTTCCCAGTCTGAACCA<br>Reverse: ATCCTGAGCTCGTCTGCTGT	NM_001190165.3
Claudin-1	Forward: CCTGCTCACCCTCATTGGAG<br>Reverse: GCTGAACTCACTCTTGGGCT	NM_001277622.1
Occludin	Forward: CCGTAACCCCGAGTTGGAT<br>Reverse: ATTGAGGCGGTCGTTGATG	NM_205128.1
ZO-1	Forward: TGTAGCCACAGCAAGAGGTG<br>Reverse: CTGGAATGGCTCCTTGTGGT	XM_413773.4
EAAT3	Forward: ACCCCCTTCTGATCACCTCT<br>Reverse: TGAGCATGCTGATTCCAAAG	XM_424930.6

GLUT2	Forward: CCGCAGAAGGTGATAGAAGC<br>Reverse: ATTGTCCCTGGAGGTGTT	NM_205129.1
PePT1	Forward: TCACTGTTGGCATGTTCCT<br>Reverse: TTCGCATTGCTATCACCTA	NM_204365.2

Abbreviations: IL-1β: interleukin-1β; IL-6: interleukin-6; IL-10: interleukin-10; IFN-γ: interferon-γ; TLR4: Toll-like receptor 4; Myd88: myeloid differentiation factor 88; NF-κB: nuclear factor kappa B p65; CAT: catalase; GSH-Px: glutathione peroxidase; SOD1: copper and zinc superoxide dismutase; Nrf2: nuclear factor erythroid 2-related factor 2; HO-1: heme oxygenase-1; NQO1: NADPH dehydrogenase 1; IGF-2: insulin-like growth factor 2; GH: growth hormone; GLP-2: glucagon-like peptide 2; ZO-1: zonula occludens 1; EAAT3: excitatory amino acid transporter 3; GLUT2: glucose transporter 2; PePT1: peptide transporter 1.
